# Bilateral total knee arthroplasty: Simultaneous or staged? A systematic review and meta-analysis

**DOI:** 10.1097/MD.0000000000015931

**Published:** 2019-05-31

**Authors:** Limin Liu, Hongtian Liu, Hui Zhang, Jingtao Song, Ling Zhang

**Affiliations:** a Department of Orthopaedic Surgery, The North China Petroleum Administration Bureau General Hospital, Renqiu; b Tuberculosis prevention and control center, Shijiazhuang Center for Disease Control and Prevention, Shijiazhuang, Hebei, P.R. China.

**Keywords:** bilateral total knee arthroplasty, meta-analysis, simultaneous, staged

## Abstract

**Background:**

Total knee arthroplasty (TKA) is one of the most successful orthopedic surgeries performed in recent decades. However, controversies still exist between conducting simultaneous or staged bilateral TKA. The objective of this study is to conduct a systematic review assessing the clinical outcome associated with simultaneous bilateral and staged bilateral total knee arthroplasty (BTKA).

**Methods:**

A search was applied to CNKI, Embase, Medline, and Cochrane central database (January 2000–July 2018). All studies that compared simultaneous bilateral TKA (simBTKA) with staged bilateral TKA (staBTKA) without language restriction were reviewed, and qualities of included studies were assessed using the Newcastle–Ottawa Scale. Data were pooled and a meta-analysis completed.

**Results:**

The 18 studies were identified to be eligible. The 18 comparative studies published from 2001 to 2018, covered 73617 participants in the simBTKA group and 61838 in the staBTKA group, respectively. Results of meta-analyses indicated that simBTKA showed a lower risk of deep infection and respiratory complications, but increased mortality, pulmonary embolism (PE), and deep-vein thrombosis (DVT) compared with staBTKA. There were no significant differences in revision, superficial infection, arthrofibrosis, cardiac complications, neurological complications and urinary complications between procedures.

**Conclusions:**

Since there are risks and benefits to both procedures, these potential complications must be interpreted in light of each individual patient's needs and concerns. Further research must be conducted, in the form of a randomized clinical trial, to evaluate the outcomes mentioned in this review.

## 1 Introduction

About 12% of adults in the US are affected by knee osteoarthritis (OA); the annual rate of total knee replacement in the US has doubled since 2000. From an economic perspective, total knee arthroplasty cost of about 10.2 billion US dollars annually.^[1]^ Total knee arthroplasty (TKA) is widely believed to be the best choice for the treatment of end-stage of knee arthropathy, and the procedure can significantly relieve pain and restore physical functioning and improve the quality of life for these patients.^[2]^ Multiple diseases such as OA, rheumatoid arthritis (RA), and hemophilia can result in severe bilateral knee destruction, with a prevalence of severe bilateral involvement as high as 19%.^[3]^ Bilateral TKA (BTKA) can be performed simultaneously under a same anesthetic or as staged procedures, with 2 unilateral knee arthroplasties under separate anesthetics and hospitalizations. Simultaneous BTKA (simBTKA) has been described as a safe and convenient procedure associated with higher patient satisfaction, faster recovery, and lower costs.^[4–6]^ Studies, however, have demonstrated higher complication rates including increased intraoperative blood loss, greater need for perioperative blood transfusion, increased rates of venous thromboembolism, cardiorespiratory complications, neurologic complications, wound breakdown, deep infection, and mortality associated with simBTKA.^[7–10]^ A staged bilateral TKA (staBTKA) may decrease the potential complication rate but has been shown to be associated with higher hospitalization costs.^[6,11,12]^

Fu et al^[7]^ conducted a meta-analysis comparing simBTKA with staBTKA, which concluded that simBTKA is associated with higher rates of mortality, blood transfusion and pulmonary embolism (PE) while decreasing the risk of revision rate and deep infection. However, the major studies included in this meta-analysis were published before 2000, and the data were obsolete, which could not reflect the current situation of the relevant indicators. With an increase in the number of studies,^[5,6,13–16]^ in patients with advance bilateral knee arthritis, simBTKA was not found to increase risk of complications such as death, cardiac complications, neurological complications, or revision compared with staBTKA.^[5,15,16]^ Re-analysis in the present study can be modified due to bias that may already exist in published studies, to show more clear results of treatment outcomes for simBTKA and staBTKA.

## 2 Materials and methods

This meta-analysis was performed by the Preferred Reporting Items for Systematic Reviews and Meta-Analyses (PRISMA)^[17]^ reporting guidelines for the conduct of meta-analysis of intervention trials.

### 2.1 Literature search

Two reviewers (LL and JS) searched CNKI (January 2000 and October 2018), Embase (January 2000 and October 2018), Medline (January 2000 and October 2018), and Cochrane central database (January 2000 and October 2018) using the search strategy of “bilateral” or “simultaneous” or “staged” or “1-staged” or “2-staged” AND “total knee arthroplasty” AND “total knee replacement”, plus “clinical trial” AND “comparative study” with no limitation of language. Additionally, we signed up with PubMed to receive automated electronic notification for any new articles containing the above search terms. Also, a manual search of references in the identified articles and systematic reviews was performed for possible inclusion.

### 2.2 Eligibility criteria

Two reviewers (HL and HZ) independently evaluated the titles and abstracts of the identified studies. Only full-text articles without language restriction were included in this meta-analysis. The following inclusive selection criteria were applied:

(1) patients had to undergo primary TKA;
(2) studies that compared simBTKA with staBTKA; and
(3) reported results including any parameters of PE, deep-vein thrombosis, revision, cardiac complications, neurologic complications, superficial infection, deep infection, and mortality, and any other systemic complications. We excluded any studies comparing bilateral to unilateral TKA, studies that evaluated other knee arthroplasties, such as resurfacing or revision TKA, and studies that assumed that unilateral TKA performed twice was 2-stage bilateral TKA.

### 2.3 Quality assessment and data extraction

The quality of the included studies was evaluated using the Newcastle–Ottawa Scale (NOS):^[18]^ based on the 3 main items: the selection of the study groups (0–4 points), the comparability of the groups (0–2 points) and the determination of either the exposure or the outcome of interest (0–3 points), with a perfect score of 9.^[19,20]^

All the data were independently and carefully extracted from the eligible studies by the same 2 reviewers (Hongtian Liu and Hui Zhang). All information relevant to the research question was extracted from the included articles including patient demographics for each treatment arm, location of the study, publication year, the incidence of complications (PE, deep-vein thrombosis, cardiac complications, neurologic complications, superficial infection, deep infection, and any other systemic complications), revision surgery rates and mortality. Any disagreement was resolved by discussion and consensus.

## 3 Statistical analysis

Odd ratios (ORs) or standard mean differences (SMDs) and corresponding 95% CI were estimated and pooled across studies to assess the discrepancy between the 2 methods with a value of *P* <.05 as significant. Heterogeneity among studies was tested by Q-test statistics with significance set at *P* <.10.^[21]^ The I^2^ statistics were used as a quantitative measure of heterogeneity, with I^2^ more than 50% indicating significant inconsistency. A random effects model was adopted to calculate pooled ORs in the case of significant heterogeneity (*P* <.10 or I^2^ >50%);^[22,23]^ otherwise, a fixed-effects model was used. The meta-analysis of significant variables was summarized graphically using a forest plot. Publication bias was assessed by Begg test and graphed by a funnel plot, a *P* <.10 was considered significant. If necessary, a sensitive analysis by excluding outlier study one by one was conducted to investigate the sources for heterogeneity. All analyses were performed using the software Stata 11.0 (Stata Corporation, College Station, TX).

## 4 Results

### 4.1 Research results and basic information

**Figure 1:**
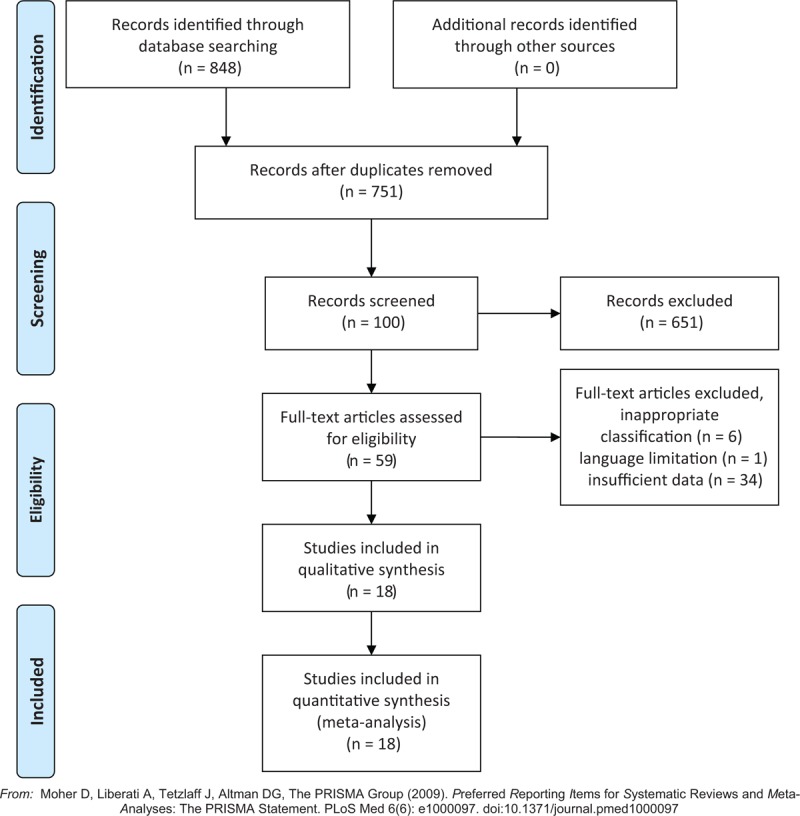
PRISMA 2009 Flow Diagram.

A total of 848 potential citations were identified; 789 were excluded due to inappropriate types (e.g., abstracts, duplicated articles, meeting reports or letters); 6 were excluded due to the inappropriate classification assessment; 1 was excluded for language limitation; 35 were excluded as they did not provide sufficient data for meta-analysis; and finally, 18 studies were identified to be eligible. The whole research procedure was presented by a flow diagram (Fig. 1). The 18 comparative studies published from 2001 to 2018, covered 73617 participants in the simBTKA group and 61838 in the staBTKA group, respectively. A summary of basic characteristics is listed in Table 1.

**Table 1:**
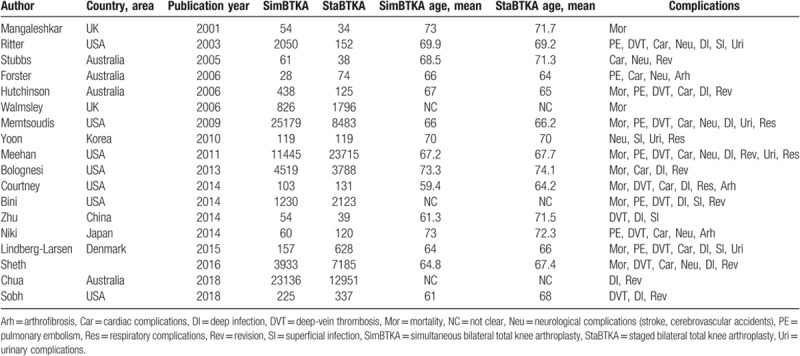
Detailed information on the basic characteristics of the 18 included studies and participants.

### 4.2 Methodological quality assessment

The outcome of methodology quality assessment was as follows: 7 studies^[5,6,15,16,24–26]^ (scored 7), mentioned that the randomization was realized by a computer-assisted tool, and allocation concealment was conducted by opaque envelope, Allocation concealment 8 studies^[14,27–33]^ (scored 6) were considered at unclear risk of bias and 3 studies^[13,34,35]^ (scored 5), which did not provide the detailed the method of randomization and sufficient data about the loss of patients.

### 4.3 Mortality

**Figure 2:**
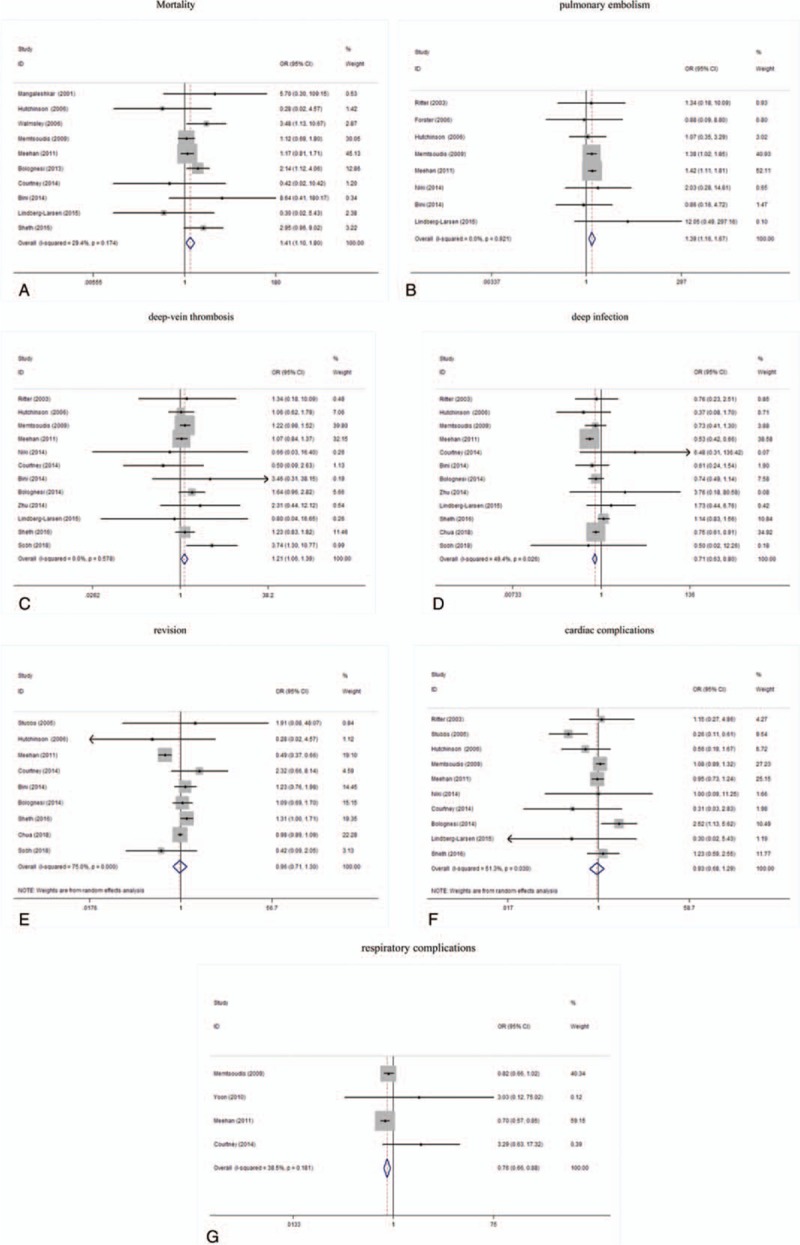
A: Forest plots of the meta-analysis of mortality. B: Forest plots of the meta-analysis of pulmonary embolism. C: Forest plots of the meta-analysis of deep-vein thrombosis. D: Forest plots of the meta-analysis of deep infection. E: Forest plots of the meta-analysis of revision. F: Forest plots of the meta-analysis of cardiac complications. G: Forest plots of the meta-analysis of respiratory complications.

**Figure 3:**
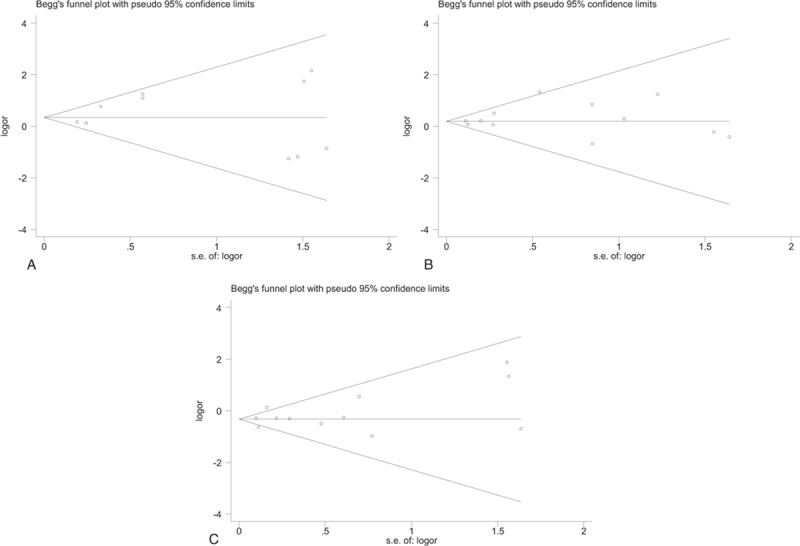
A: Begg funnel plot for publication bias investigated no differences of mortality between simBTKA and staBTKA group (*P* = .592). B: Begg funnel plot for publication bias investigated no differences of DVT between simBTKA and staBTKA group (*P* = .945). C: Begg funnel plot for publication bias investigated no differences of deep infection between simBTKA and staBTKA group (*P* = 1).

Ten studies^[5,15,16,24–27,30,33,35]^ including 92,782 patients were included for analysis of mortality. The prevalence of mortality was significantly higher in patients that had undergone simBTKA when compared with those who had undergone staBTKA (OR 1.41, 95% CI 1.10–1.18), consistent with low heterogeneity (*P* = .174, I^2^ = 29.4%; Table 2; Fig. 2A). Begg funnel plot for publication bias investigated no differences of mortality between simBTKA and staBTKA group (*P* = .592; Fig. 3A).

**Table 2:**
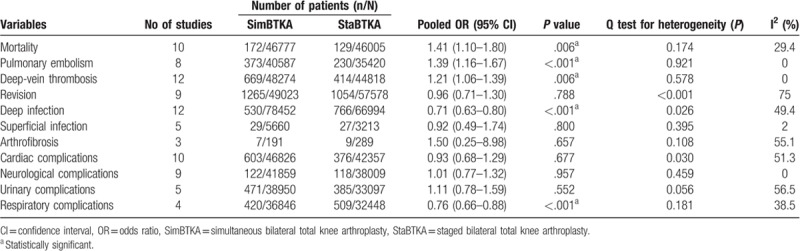
Detailed data on comparing variables between both methods and the outcomes.

### 4.4 Pulmonary embolism

A total of 8 studies^[15,16,25,27,28,30,32,34]^ reported the PE and the meta-analysis of these studies showed that patients in simBTKA group appeared more prone to PE (OR 1.39, 95% CI 1.16–1.67), consistent with no heterogeneity (*P* = .921, I^2^ = 0; Table 2; Fig. 2B).

### 4.5 Deep-vein thrombosis

Twelve studies^[5,6,13,15,16,24–28,30,32]^ reported deep-vein thrombosis (DVT). Using a fixed-effects model, patients in simBTKA group did have a significantly higher rate of DVT (OR 1.21; 95% CI 1.06–1.39; Table 2; Fig. 2C), with no significant heterogeneity. Begg funnel plot for publication bias investigated no differences of DVT between simBTKA and staBTKA group (*P* = .945; Fig. 3B).

### 4.6 Deep infection and superficial infection

Twelve studies^[5,6,13–16,24–27,30,32]^ involving 78452 simBTKAs and 66994 staBTKAs reported the incidence of postoperative deep infection. The rate of deep infection was 0.7% in the simBTKA group and 1.1% in the staBTKA group, with significant difference (OR 0.71, 95% CI 0.63–0.80) without heterogeneity (*P* = .026, I^2^ = 49.4%; Table 2; Fig. 2D). Begg funnel plot for publication bias investigated no differences of deep infection between simBTKA and staBTKA group (*P* = 1; Fig. 3C). Simultaneously, 5 studies^[13,15,16,29,32]^ reported the incidence of postoperative superficial infection,which was 0.5% in the simBTKA group and 0.8% in the staBTKA group. There were no significant differences (OR 0.92, 95% CI 0.49–1.74; Table 2).

### 4.7 Revision

Nine studies^[5,6,14,16,24,26,27,30,31]^ reported the incidence of revision rate, with 2.6% of the simBTKA group and 1.8% of the staBTKA and the pooled results for meta-analysis suggested no significant difference (OR, 0.96; 95% CI, 0.71–1.30; I^2^ = 75%; Table 2; Fig. 2E). After sensitive analyses, heterogeneity was resolved and the significance did not change (Table 3).

**Table 3:**
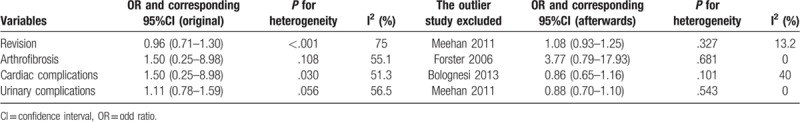
Results of sensitive analysis for variables.

### 4.8 Cardiac complications

Ten studies^[5,15,24–28,30–32]^ reported the cardiac complications, with no significant difference between simBTKA group and staBTKA group (OR, 0.93; 95% CI, 0.68–1.29) with heterogeneity (I^2^ = 51.3%) (Table 2; Fig. 2F). After sensitive analyses, heterogeneity was resolved and the significance did not change (OR, 0.86; 95% CI, 0.65–1.16; I^2^ = 40%; Table 3).

### 4.9 Respiratory complications

There were 4 included studies^[24,25,29,30]^ reported respiratory complications. The meta-analysis showed there was significant difference between simBTKA group and staBTKA group (OR, 0.76; 95% CI, 0.66–0.88; Table 2; Fig. 2G), with no evidence of heterogeneity (*P* = .181, I^2^ = 38.5%; Table 2).

However, with regard to neurological complications (OR, 1.01; 95% CI, 0.77–1.32), urinary complications (OR, 1.11; 95% CI, 0.78–1.59), there were no significant differences observed between both 2 methods. The results are presented in Table 2.

## 5 Discussion

OA is the most common musculoskeletal disease^[36,37]^ causing significant disability.^[38]^ With the increasing life expectancy and predicted rise in the prevalence of obesity, the numbers affected by OA are likely to keep on increasing.^[39]^ The gold standard for the treatment of end-stage OA is TKA,^[40]^ which is now one of the most common procedures performed by orthopedic surgeons. However, controversy arises when a patient presents with bilateral degenerative disease: simBTKA or staBTKA? We, therefore, performed this systematic and meta-analysis concerning the comparison of simBTKA and staBTKA techniques. The main finding of the present study was that simBTKA translates into complete elimination of the disease in a single hospital admission, lower incidence of deep infection and respiratory complications. Meanwhile, staBTKA has been associated with lower rates of mortality, PE and deep-vein thrombosis.

### 5.1 Mortality

Our results reveal a combined mortality rate of 0.32% for simBTKA (0.37%) and staBTKA (0.28%). Previous studies have also reported a combined mortality rate between 0.16% and 0.77%.^[5,15]^ Some studies have shown increased mortality rate for simBTKA compared with staBTKA.^[26,33]^ A number of more recent studies have shown no difference in mortality between simBTKA and staBTKA,^[16,24]^ which may correlate with the improvement in surgical technique over time or better patient selection, although some had relatively small numbers. In the present study, the reason why the incidence of mortality was significant higher in simBTKA group was might be influenced by the higher rate of PE and DVT.

### 5.2 Deep-vein thrombosis and PE

Venous thromboembolic events (VTEs) are a common and potentially fatal complication in patients undergoing TKA. Previous studies have shown that the rate of VTE in patients undergoing TKA without any prophylaxis can be as high as 88%.^[41,42]^ Meehan et al^[30]^ reviewed the California Patient Discharge database who underwent bilateral TKA between 1997 and 2007. Of those, 11445 patients underwent simBTKA and 23715 patients had undergone staBTKA. They found that the probability of a DVT developing in the first 2 months after simBTKA and staBTKA was 0.86% and 0.81%, respectively. Niki et al^[28]^ reported the DVT rate of patients undergoing simBTKA was 0 while the rate for patients undergoing staBTKA was 0.84%. According to our results, the simBTK group was 1.21 times more at risk of developing DVT than patients undergoing staBTKA. The patients with simBTKA (0.92%) had a higher proportion of patients with PE than the patients who had surgery on both knees at separated time (0.65%). The fact that PE was the dominating cause (40%) of death in the simBTKA group and a considerably less common cause of death in the staBTKA group suggests the increased embolic load during simBTKA causes increased mortality. The use of a pneumatic tourniquet, intramedullary guides, and cement are factors that probably are of importance. Larson et al^[43]^ report a PE rate of 0 in patients undergoing simBTKA treated with 650 mg aspirin twice daily combined with a knee-high intermittent compression device. Consequently, prophylaxis has become the standard of care following TKA^[44]^ which can reduce the rate of VTE, including DVT or PE. Prophylaxis can be achieved with the use of chemotherapeutic agents or mechanical devices.^[45]^

### 5.3 Infection and revision rate

A deep infection was considered any infection that occurred inside the knee joint and required arthrotomy, liner removal, debridement, synovectomy, or even revision knee arthroplasty. A superficial infection was any infection of the skin that responded well to antibiotics with no residual issues. Previous studies^[30,32,46]^ showed that staged procedures and longer hospitalization were significant predictors for prosthetic joint infection. In this meta-analysis, the incidence of peri-operative infection events in the superficial (0.84% vs 0.51%) and deep infection (1.14% vs 0.68%) subgroups were higher in the staBTKA group. Superficial surgical infections increase the duration of a patient's hospital stay and may lead to periprosthetic joint infection.^[47]^ Simultaneously, simBTKA cases have longer surgical times, but lower operative time cumulatively, which can reduce the rate of deep infection. The overall revision rate was 2.58% in the simBTKA cohort compared with 1.83% in the staBTKA. Coupled with the finding that the incidence of deep infection after simBTKA did differ significantly from the incidence after staBTKA, these findings strongly suggest that the risk of deep infection is not a function of the number of joints revision.

### 5.4 Cardiac complications

Cardiac complications such as arrhythmias, myocardial infarction, and congestive cardiac failure are some of the common reported after simBTKA.^[10,27,48]^ The cause of this remains unclear, however, the rates of cardiac complication are reported to be higher in patients with pre-existing comorbid medical conditions and in the elderly patients (>80 years). It can be postulated that the physiological stress imposed by the simultaneous procedure on this group of high-risk patients with presumed suboptimal cardiorespiratory reserve could be the cause of increased complications.^[49]^ In Niki's study,^[28]^ shorter operative time and reduced blood loss would have substantially contributed to decreased incidence of cardiovascular complication after simBTKA. The present study reported the incidence of cardiac complications, with 1.3% of the simBTKA group and 0.9% of the staBTKA and the pooled results for meta-analysis suggested no significant difference. Nevertheless, the higher risk of cardiac complications following previous comorbidity calls for caution in the selection of patients for simBTKA.

### 5.5 Neurological complications

A higher rate of postoperative neurological complications in the simultaneous bilateral group could be partly explained by a number of factors, including increased postoperative blood loss, increased hypoxemia and anemia, increased need for analgesics, and increased fluid shifts and potential electrolyte imbalances. However, neurological complications, together with the increased demand for nasal oxygen, could be attributed to increased systemic dissemination of fat from the displacement of intramedullary fat intraoperatively. Several authors have shown that bilateral procedures result in an increased prevalence of fat emboli with resulting neurological and pulmonary effects.^[50–52]^ Nevertheless, in this meta-analysis, we found that a simultaneous procedure had no increased risk of neurological change over a staged procedure. This conflict might be explained by improvements in surgical techniques or lacking exact definition of neurological complications in each previous study.

We also observed higher incidence of respiratory complications in the staBTKA group (1.6% vs 1.1%). Patients who had chronic obstructive pulmonary diseases, adult respiratory distress syndrome or the other chronic lung disease had a high risk of respiratory complications among staBTKA recipients. This is consistent with previous studies which showed that staged procedures and longer hospitalization are significant predictors for respiratory complications.^[25,29]^

In clinical practice, we prefer bilateral staged total knee arthroplasties to bilateral simultaneous knee arthroplasty in elderly patients with moderate to severe symptoms of both knees for treatment of arthritis. There is evidence of improved knee functional outcomes and economic benefits in patients underwent simBTKA compared to staBTKA, however, these advantages would not be justified when considering the incidence of major postoperative complications and mortality.

Some limitations in this meta-analysis have to be mentioned. First, a weakness exists in the analyses, in which not all the ORs regarding the potential complications applied for the meta-analysis were adjusted because a lot of reports could only provide the univariate rather than multivariate statistics. Likewise, some studies might choose not to report insignificant results or results of no interest, potentially resulting in a considerable amount of missing data. Hence, our overall effect may be somewhat an overestimate. Second, most of the included studies were observational and therefore with inevitable recall and interviewer biases, which might affect the associations between the simBTKA group and staBTKA. Third, the measurements of various factors differed from each other, and follow-up periods ranged widely from several months to several years. Therefore, a significant heterogeneity was unavoidable in this review. However, after sensitive analyses, heterogeneity was resolved (I^2^ <50%), showing analyses were robust and the results reliable. Fourth, there might be operator-dependent and append subjective factors in the quality of assessment process. Nevertheless, the 2 reviewers evaluated the identified studies independently and any disagreement was resolved by discussion and consensus. Although this meta-analysis investigates some higher incidence of complications after bilateral total knee arthroplasty, we should treat these results cautiously on the background of potential defects, and more research studies with larger sample size and better design should be conducted.

Although some limitations were unavoidable, this study has some merits. First, the search style based on the computer and manual search ensures a complete inclusion of relevant studies. Second, no significant heterogeneity was observed in most variables except for the item of revision and cardiac complications; even so, heterogeneity was diminished using sensitivity analysis and this did not alter the result.

## 6 Conclusion

In summary, if patients have bilateral knee disease, simBTKA had a lower risk of deep infection and respiratory complications, but associated with higher rates of mortality, PE, and DVT compared with staBTKA. However, this study does not encourage performing simultaneous over staged bilateral TKA. Since there are risks and benefits to both procedures, these potential complications must be interpreted in light of each individual patient's needs and concerns. Further research must be conducted, in the form of a randomized clinical trial, to evaluate the outcomes mentioned in this review.

## Author contributions

**Data curation:** Hongtian Liu, Hui Zhang.

**Formal analysis:** Ling Zhang.

**Investigation:** Ling Zhang.

**Methodology:** Hui Zhang.

**Software:** Hongtian Liu, Hui Zhang.

**Supervision:** Limin Liu.

**Writing – original draft:** Jingtao Song.

**Writing – review & editing:** Limin Liu.
